# Antibacterial action and target mechanisms of zinc oxide nanoparticles against bacterial pathogens

**DOI:** 10.1038/s41598-022-06657-y

**Published:** 2022-02-16

**Authors:** Carolina Rosai Mendes, Guilherme Dilarri, Carolina Froes Forsan, Vinícius de Moraes Ruy Sapata, Paulo Renato Matos Lopes, Peterson Bueno de Moraes, Renato Nallin Montagnolli, Henrique Ferreira, Ederio Dino Bidoia

**Affiliations:** 1 Department of General and Applied Biology, Sao Paulo State University (UNESP), 24-A Avenue, 1515, Rio Claro, SP 13506-900 Brazil; 2 College of Technology and Agricultural Sciences, Sao Paulo State University (UNESP), SP‑294, km 651, Dracena, SP Brazil; 3 School of Technology, State University of Campinas (UNICAMP), 6122, Limeira, SP 13083-970 Brazil; 4 Department of Natural Sciences, Mathematics and Education, Agricultural Sciences Centre, Federal University of Sao Carlos (UFSCar), SP-330, km 174, Araras, SP Brazil

**Keywords:** Applied microbiology, Nanoparticles, Bacteria

## Abstract

Zinc oxide nanoparticles (ZnO NPs) are one of the most widely used nanoparticulate materials due to their antimicrobial properties, but their main mechanism of action (MOA) has not been fully elucidated. This study characterized ZnO NPs by using X-ray diffraction, FT-IR spectroscopy and scanning electron microscopy. Antimicrobial activity of ZnO NPs against the clinically relevant bacteria *Escherichia coli*, *Staphylococcus aureus*, *Pseudomonas aeruginosa*, and the Gram-positive model *Bacillus subtilis* was evaluated by performing resazurin microtiter assay (REMA) after exposure to the ZnO NPs at concentrations ranging from 0.2 to 1.4 mM. Sensitivity was observed at 0.6 mM for the Gram-negative and 1.0 mM for the Gram-positive cells. Fluorescence microscopy was used to examine the interference of ZnO NPs on the membrane and the cell division apparatus of *B. subtilis* (*amy*::pspac-ftsZ-gfpmut1) expressing FtsZ-GFP. The results showed that ZnO NPs did not interfere with the assembly of the divisional Z-ring. However, 70% of the cells exhibited damage in the cytoplasmic membrane after 15 min of exposure to the ZnO NPs. Electrostatic forces, production of Zn^2+^ ions and the generation of reactive oxygen species were described as possible pathways of the bactericidal action of ZnO. Therefore, understanding the bactericidal MOA of ZnO NPs can potentially help in the construction of predictive models to fight bacterial resistance.

## Introduction

Nanoparticles (NPs) of metal oxide stand out in the field of antimicrobial compounds by their catalytic inhibition activity^1,2^. However, their bactericidal mechanism of action (MOA) depends on several parameters, such as their morphology, composition and concentration^3,4^. Zinc oxide (ZnO), magnesium oxide (MgO) and titanium dioxide (TiO_2_) are substances recognized as safe when used as food additives or drug deliverers according to the Food and Drug Administration (FDA 2011) US Code of Federal Regulations (Title 21-CFR 182.8991)^5^. Zinc oxide nanoparticles (ZnO NPs) are the most promising inorganic materials that have bactericidal action and can be found in the composition of pharmaceutical drugs, sanitizers, cosmetics and food packaging processes^6^. However, the targets of ZnO NPs in bacteria of clinical importance are not fully understood. Zanet et al.^7^ carried out experiments using ZnO NPs against the model cell *Saccharomyces cerevisiae* in order to elucidate the main MOA, and concluded that the effects of ZnO NPs depend on their composition and dose.

The synthesis of ZnO NPs can be achieved by chemical precipitation^8^, salt reduction^9^, sol–gel way based on an acetate precursor^10^, and sonochemical synthesis^11^. However, different synthetic pathways yield ZnO particles with variable morphologies and sizes^9,12,13^. Thus, their MOA, as well as their interaction with diverse cell structures, may vary significantly.

ZnO is a transition metal oxide and semiconductor with high binding energy which allows for a highly oxidative character^14^. This reaction leads to the formation of reactive oxygen species as the pathway of bactericidal action. In addition, another bactericidal MOA occurs through the release of zinc ions (Zn^2+^) that damage the cell membrane and may interrupt some metabolic pathways^15^. Thus, additional studies about the antibacterial MOA of ZnO NPs can relevantly contribute to the prediction of possible mechanisms of bacterial resistance and for the optimizing of the contact time and effective inhibition action.

Cell division is a critical process for microbial survival. Among the proteins involved in this process, FtsZ has a pivotal function in which it serves as a scaffold for the assembly of a multiprotein complex structure, the divisome, responsible for coordinating all the steps of cell division and cell wall remodeling^16^. The protein acts during cell division as an organizer of the cytoplasmic ring in bacteria and can be considered the main target of several bactericidal compounds^17^. Bactericidal agents act in diverse ways, such as inhibition of FtsZ in the cell division pathway, and can be identified by cytological profile of cells expressing FtsZ-GFP and observed by fluorescence microscopy^18^.

In this study, the model Gram-positive bacteria *Bacillus subtilis* (ATCC 19659) and a set of clinically relevant bacteria *Escherichia coli* (ATCC 8739), *Staphylococcus aureus* (ATCC 6538), and *Pseudomonas aeruginosa* (ATCC 27853) were used to determine the inhibitory concentration of ZnO NPs and to evaluate their effect on the bacteria cytological profile. The focus of the present study was to evaluate the action of ZnO NPs on cell morphology, chromosome organization, and protein production. Therefore, the cytoplasmatic membrane and other proteins, such as FtsZ, which forms the scaffold for the divisome, were not included in the spectra of analysis. *Bacillus subtilis* FtsZ was used to evaluate any interference in the formation of the FtsZ ring.

## Materials and methods

### Synthesis of ZnO NPs

All reagents were purchased from Sigma-Aldrich (Taufkirchen, Germany). ZnO NPs were synthesized by sonochemical-coprecipitation of 2 mM solution of zinc chloride followed by dripping it with ammonium hydroxide^19^. Next, the mixture was heated to 60 °C under continuous stirring until complete precipitation. The precipitate underwent ultrasonic bath sonicator (USC 1400) for 30 min to obtain NPs, followed by vacuum filtration using a 0.22 μm cellulose membrane, washing with deionised water and finally drying at 100 °C overnight.

### Characterization of the synthesized nanomaterial

ZnO NPs were characterized using Fourier transform infrared spectrophotometer FT-IR (Shimadzu Model 8300), adjusted for scanning at 4000–400 cm^−1^. For the analysis, a KBr pellet was made with the nanomaterial sample^20^. Micrographs of ZnO NPs were taken by Scanning Electron Microscope (SEM)—JEOL JSM-IT100 operated at 30 kV coupled to a Bruker Quantax Energy Dispersive Detector (EDS), in order to study the morphological characteristics. The samples were coated with a gold layer by a metalization process before SEM readings. Finally, the crystalline structure of the ZnO NPs was characterized by X-ray diffraction powder (XRD, PHILIPS, X’ pert-MPD system) using Cu Kα radiation (*λ* = 1.5418 Å). The X-ray wavelength was 0.15418 nm and the diffraction patterns were measured in the range of 2*θ* from 20° to 65°.

### Bacterial strain and growth conditions

*Escherichia coli* (ATCC 8739), *Staphylococcus aureus* (ATCC 6538), *Bacillus subtilis* (ATCC 19659), *Pseudomonas aeruginosa* (ATCC 27853) and the mutant *Bacillus subtilis* (*amy*::*pspac*‐*ftsZ*‐*gfpmut1*; a gift of Dr. F. Gueiros-Filho, IQ, USP, São Paulo, Brazil) expressing FtsZ‐GFP were cultivated in nutrient broth medium (5 g L^−1^ of peptone; 3 g L^−1^ of beef extract; for solid medium was added 15 g L^−1^ of bacterial agar) at 28 °C for 24 h in shaker at 200 rpm. All reagents for cellular growth were purchased from Himedia Laboratories Ltd. (Mumbai, India).

### Antibacterial activity assay

The antibacterial activities expressed as Inhibitory Concentrations (ICs) of the ZnO NPs were determined by using the Resazurin Microtiter Assay (REMA)^21^. ZnO NPs at concentrations between 0.2 and 1.4 mM were placed in 96-well microplates. The strains were inoculated to independent trials at a final concentration of 10^5^ cells per 100 μL in each well and incubated for 12 h at 30 ± 1 °C. Nisin at 5 µg mL^−1^ and Kanamycin at 20 µg mL^−1^ from Sigma-Aldrich (Taufkirchen, Germany) were used as reference antibiotic (positive controls) against Gram-positive and Gram-negative bacteria, respectively. Nutrient broth medium was used as negative control. After the incubation period, the inhibition of cell growth was measured by the addition of 0.1 mg mL^−1^ resazurin (Sigma-Aldrich; Taufkirchen, Germany) in each well. In live cells, resazurin is reduced to resorufin (a fluorescent compound) in the presence of NADH, which indicates cell activity^18^. The fluorescence intensity of resorufin was detected in a plate reader (Synergy H1N1—BioTek, Winooski, VT, USA) set to the excitation and emission wavelengths of 530 and 590 nm, respectively. The results of this assay were used to plot the correlation between ZnO NPs concentration and the inhibition of cell growth. Non-linear regression models were used to derive the IC_100_ values for each bacterial strain (100% inhibitory concentration).

### Effect of ZnO NPs on the membrane integrity

*E. coli*, *P. aeruginosa*, *B. subtilis* and *S. aureus* at 10^5^ cells were exposed to ZnO NPs at concentrations equivalent to their IC_100_ in 100 μL of media for 15 min. Next, 900 μL of phosphate buffer were added to stop the reactions. Next, cells were stained using 0.01 mg mL^−1^ propidium iodide (PI) and 0.02 mg mL^−1^ DAPI (4′,6-diamidino-2-phenylin-dole). DAPI stains the nucleoid of every cell, whereas propidium iodide (PI) is a nucleic acid dye that penetrates only cells with damaged cytoplasmatic membranes. Untreated cells were used as negative control, while positive control for damaged membranes was generated by heat-shock stress (Gram-negative) and Nisin treatment (Gram-positive). *B. subtilis* (*amy*::*pspac*‐*ftsZ*‐*gfpmut1*) expressing FtsZ-GFP was used to investigate the potential of the compound to interfere with the divisional septa. The bacterial cells were cultivated in the presence of 0.02 mM Isopropyl β‐d‐thiogalactopyranoside (IPTG) to induce the expression of FtsZ‐GFP from the *pspac* promoter. Next, 100 µL of the cultures (adjusted to contain ~ 10^5^ cells) were exposed for 15 min to ZnO NPs at its respective IC_100_. Cells were washed with water and resuspended in 100 µL of 0.85% NaCl solution prior to microscope observation^18^. Cells were immobilized onto agarose-covered slides and visualized using an Olympus BX-61 (Tokyo, Japan), equipped with a monochromatic camera OrcaFlash 2.8 (Hamamatsu, Japan). Images were processed by the software CellSens version 11 (Olympus). One hundred cells were considered (n = 100) per treatment for quantifications.

## Results and discussion

### Characterization of ZnO NPs

The XRD peaks were consistent with ZnO crystallite. The analysis showed no extra peaks, which is due to the purity of the material applied during the synthesis of ZnO NPs. The positions of the diffraction peaks showed the same pattern found in the Joint Committee on Powder Diffraction Standards: 36–1451 database (JCPDS).

**Figure 1:**
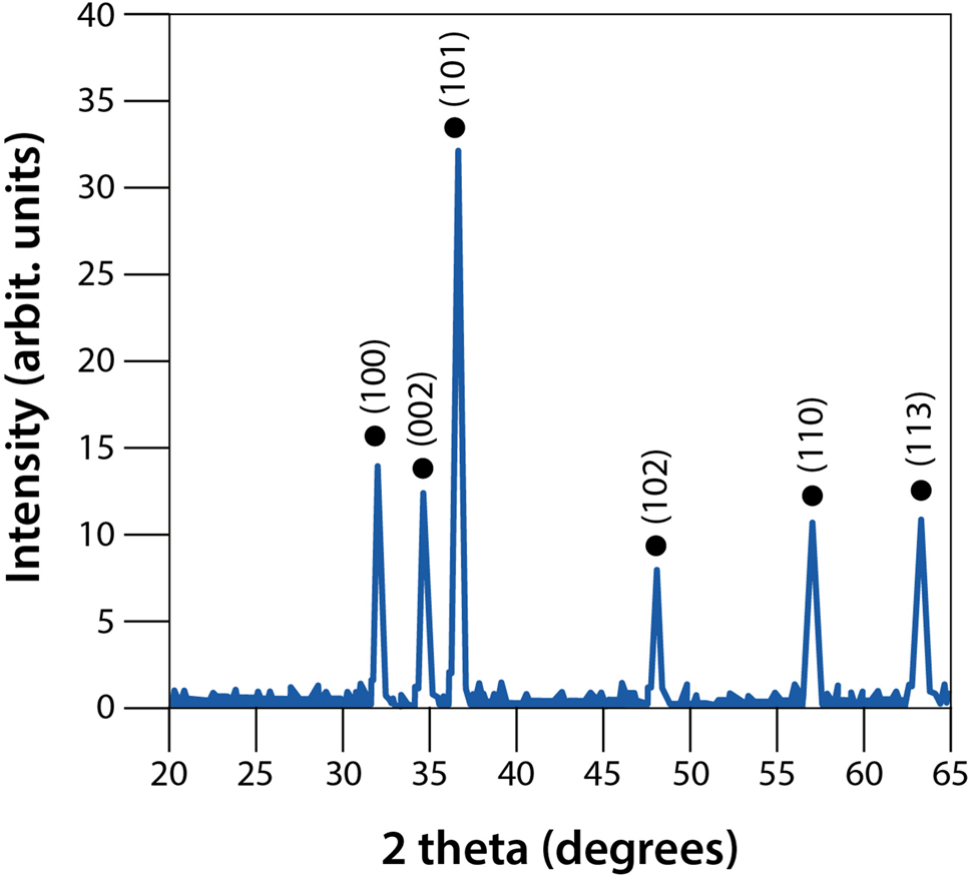
XRD power of ZnO NPs.

Figure 1 shows the diffraction peaks of ZnO NPs at (100), (002), (101), (102), (110), (103) which correspond respectively to the values in degrees (2θ) at 31.34°, 34.50°, 36.32°, 47.60°, 56.68°, 62.94°. High diffraction peaks indicated the crystalline nature of the material^22^.

Table 1 shows the values of the structural parameters used to calculate the size of the ZnO crystallite by Eq. (1)^23^. The high intensity peak at (101) was used to determine the lattice parameters.

1 $$D=\left[\frac{0.9\lambda }{\beta \cos \theta }\right] \times 100$$

where *D* is the size of the ZnO crystallite; *λ* is the wavelength of Cu Kα radiation at 1.5418 Å; *θ* is the Bragg diffraction angle, and *β* is the full width at half maximum intensity of the diffraction peak of the sample.

**Table 1:** Structural parameters of ZnO crystallite.

Lattice parameters *a* (Å)	Lattice parameters *c* (Å)	*c*/*a* ratio	Volume of unit cell (Å^3^)	Average crystallite size (nm)	Microstrain *ε* (× 10^3^)
3.24	5.21	1.608	47.48	82.38	0.47

The crystallite size can be measured more accurately by high resolution X-ray diffraction (HRXRD) using the Bond method, which increases peak resolution to find the values of the Lattice parameters^23,24^. In this study, we determined by XRD powder that most synthesized ZnO crystallites are around 80 nm in size. Similar results obtained through the synthesis of ZnO NPs by sonochemical–coprecipitation were shown by Khataee et al.^25^.

**Figure 2:**
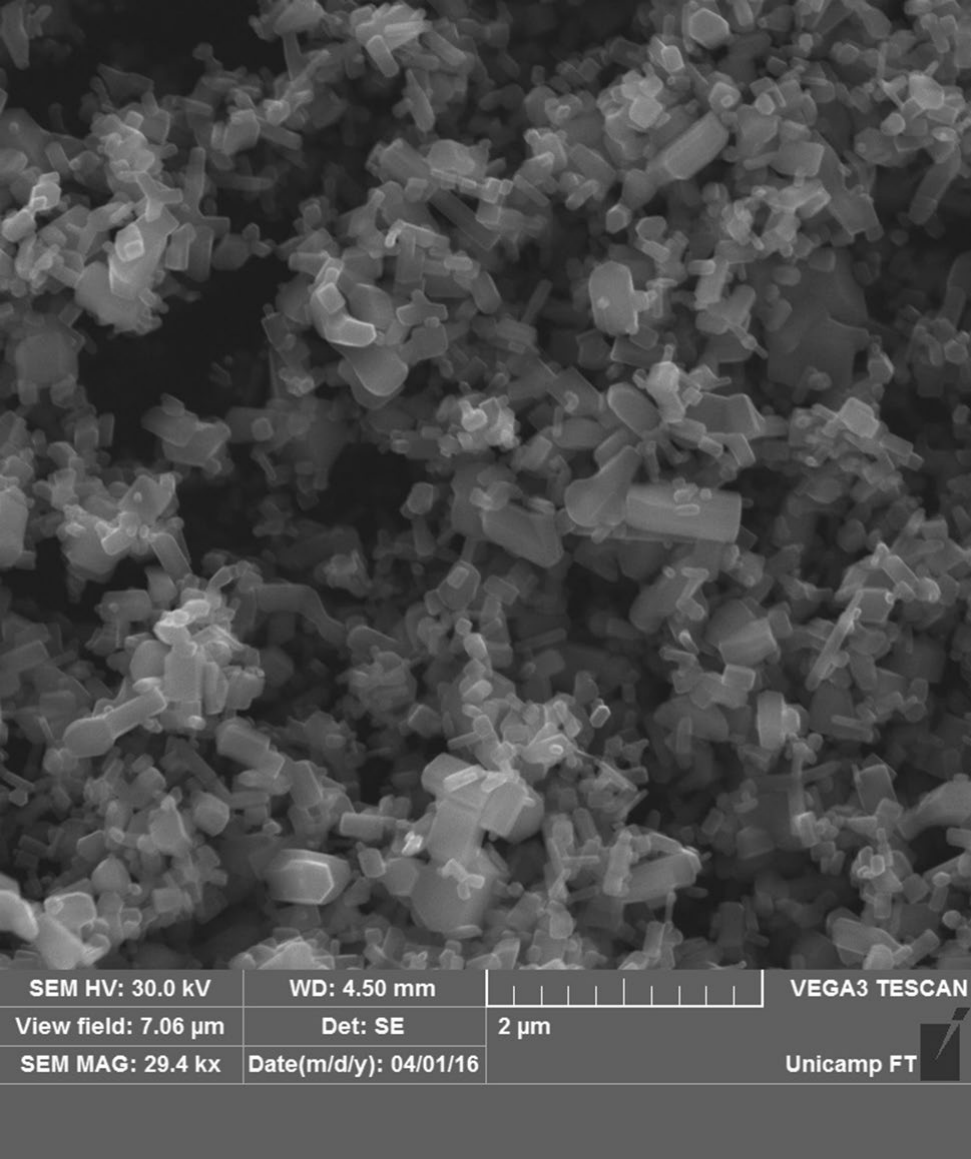
Surface morphology of ZnO NPs by SEM. 2 µm scale bar; 29.0 kx magnification.

The surface appearance and morphology of synthesized ZnO NPs were analyzed by SEM at 29.0 kx. Based on the images in Fig. 2, the ZnO NP showed complex bead and rod morphology. In addition, the ZnO NPs have an irregular size with formation of aggregated nanocrystallite.

**Figure 3:**
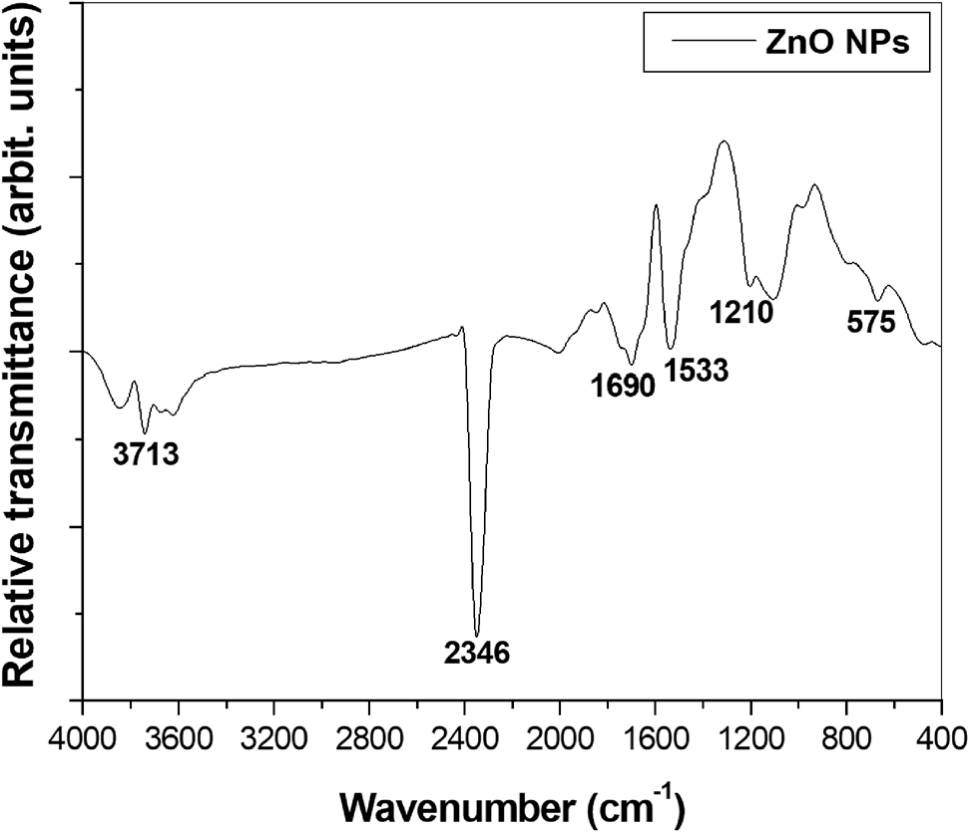
FT-IR spectrum of ZnO NPs.

FT-IR spectra are like molecular fingerprints that provide a valuable insight into chemical structures and their changes due to interactions with other molecules^20^. FT-IR analyses detected the characteristic functional groups associated with the ZnO NPs (Fig. 3). The peak at 575 cm^−1^ corresponds to the stretching/vibration of the metal–oxygen bond in Zn–O. The peak at 3713 cm^−1^ corresponds to carbon residues identified during the sample measurement; and 1210 cm^−1^ belongs to the elongation of C–O. Hydrogen bonds are displayed at 1690 and 2346 cm^−1^, and they are ascribed to the stretching vibration of hydroxyl compounds. The hydroxyl group influences photocatalytic reactions in ZnO by generating superoxide radicals, which act as an antimicrobial^26^.

### Antimicrobial activity

**Figure 4:**
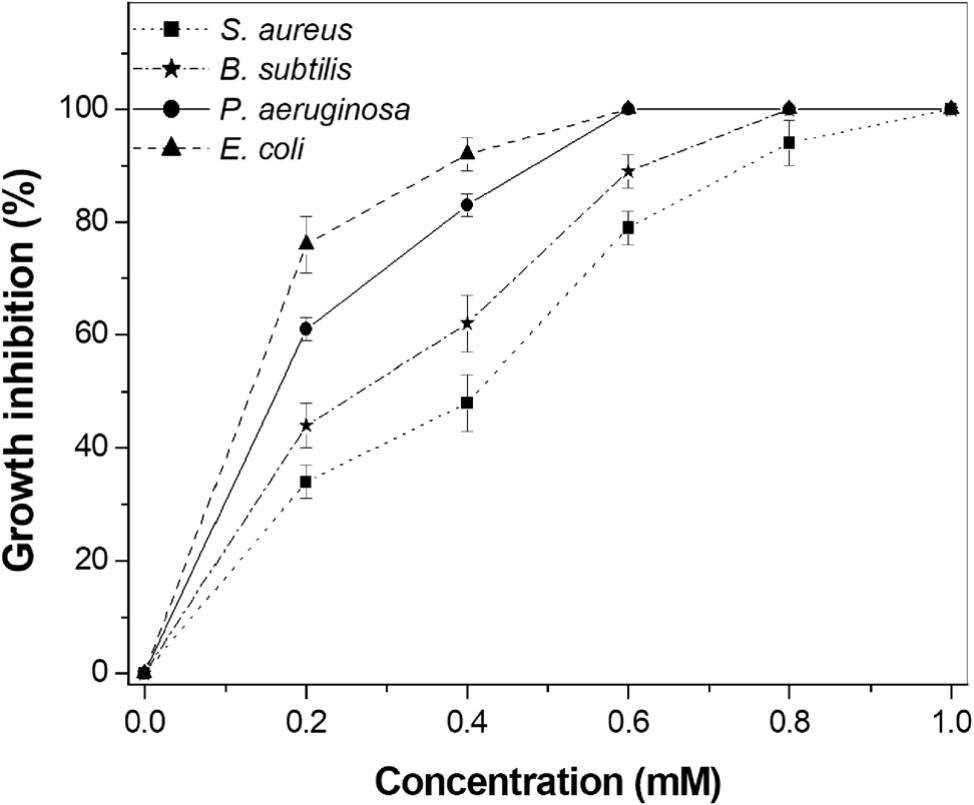
Antimicrobial activity of ZnO NPs determined by REMA.

The bactericidal activities of ZnO NPs against *E. coli*, *P. aeruginosa*, *S. aureus* and *B. subtilis* were evaluated by monitoring cell respiration. Polynomial regression applied to the dose–response data was used to extrapolate the IC_100_ values, which were expressed as mM. The decrease in cell numbers observed after treatment is shown in by the plot containing the concentration of ZnO NPs versus the inhibition of cell growth (Fig. 4).

ZnO NPs inhibited growth of *E. coli* and *P. aeruginosa* with IC_100_ values of 0.6 mM for both strains. The IC_100_ values for *B. subtilis* and *S. aureus* were estimated at 0.8 and 1.0 mM, respectively. Gram-negative bacteria have a thin layer of peptidoglycan between two membranes, which is known to provide antimicrobial resistance^27^. In addition, dissociated carboxyl groups present in the membranes generate negative charges on the cell surface. ZnO NPs, on the other hand, have a positive charge, with a zeta potential of + 24 mV^28^. As a result of electrostatic forces, damage to the cell membrane occurs due to electrostatic gradient differences across the negative membrane and the positive charges of the Zn^2+^ ions. Therefore, *E. coli* and *P. aeruginosa* died with the lowest concentration of ZnO NPs. Although the present study did not observe a large difference in the IC values for Gram-negative and Gram-positive, it is noteworthy that Gram-positive exhibited IC_100_ values higher than Gram-negative. Similar inhibition in Gram-negative bacteria was previously reported by Yusof et al.^29^, Saqib et al.^30^ and Zubair and Akhtar^31^; however, with slight variations in the IC_100_ values due to differences in the synthesis of the nanomaterial, which yields unique characteristics to each one of them. Overall, the results were close; however, our study not only investigated the percentage of inhibitory growth, but also the MOA target of ZnO NPs in clinical strains.

### Effect of ZnO NPs on the bacteria cell

Bacterial cell division is a complex and dynamic process, which starts with the polymerization of the FtsZ protein in order to assemble the divisome, which will guide all the processes related to cell division and cell wall synthesis and remodelling^32^. FtsZ is the ancestral tubulin conserved in bacteria, which exerts its function dependent on the nucleotide guanosine triphosphate (GTP)^33,34^. Some bactericidal compounds act by preventing the GTPase activity of FtsZ, which will inhibit cell division and lead to cell death^17^. In addition, blockage of the cell division process generally leads to cell filamentation, which can be easily accessed by fluorescence microscopy. Alternatively, by using mutant cells expressing labelled division proteins, e.g. FtsZ-GFP, one can follow the dynamics of division and study the effects compounds might have on the process.

**Figure 5:**
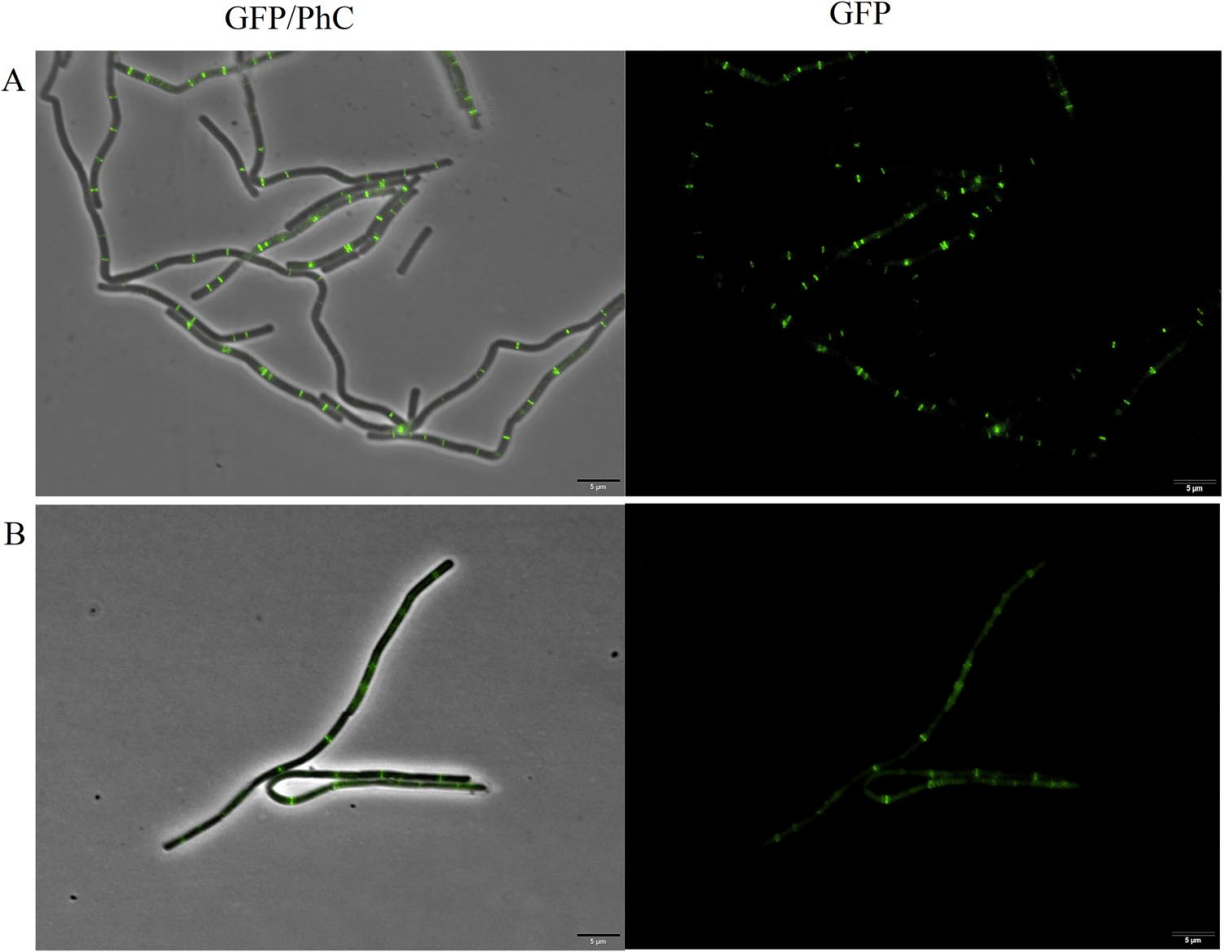
*B. subtilis* expressing FtsZ-GFP. (**A**) Control of cells grown in nutrient medium and diluted to 10^6^ cells per mL^−1^. (**B**) Cells after 15 min of exposure to ZnO NPs in the IC_100_. GFP/PhC is the phase contrast images superimposed on the GFP fluorescence images. Scale bar 5 μm; × 100 magnification.

*B. subtilis* expressing FtsZ‐GFP was exposed to ZnO NPs at its IC_100_ for 15 min, and afterwards observed under the microscope (Fig. 5). Note that even after ZnO NPs exposure, the cells still have intact bars perpendicular to the long axis of the rods, which is the normal profile for the Z-ring. This cytological profile was comparable to the control and did not show any disruption of the divisional ring.

**Figure 6:**
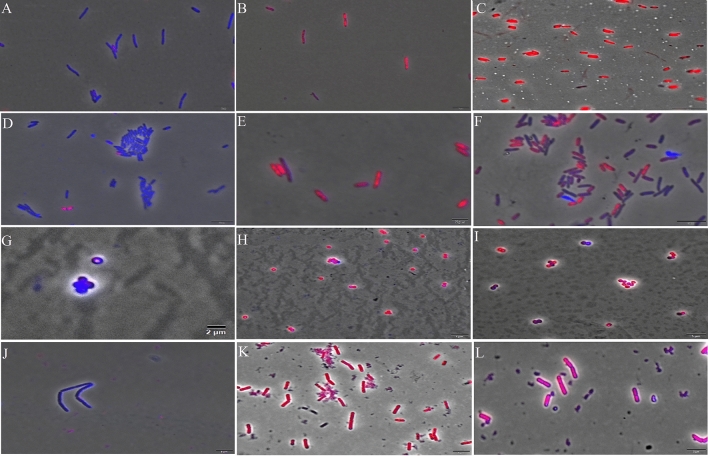
Fluorescence microscopy in cells stained with DAPI and PI after 15 min of exposure to ZnO NPs. Cells with intact membranes are artificially stained in blue, while cells with damaged membranes are stained in red. (**A**) *E. coli* (ATCC 8739) cells in nutrient broth medium (negative control); (**B**) *E. coli* (ATCC 8739) cells treated with heat-shock stress (positive control); (**C**) *E. coli* (ATCC 8739) cells treated with ZnO NPs at IC_100_; (**D**) *P. aeruginosa* (ATCC 27853) cells in nutrient broth medium (negative control); (**E**) *P. aeruginosa* (ATCC 27853) cells treated with heat-shock stress (positive control); (**F**) *P. aeruginosa* (ATCC 27853) cells treated with ZnO at IC_100_; (**G**) *S. aureus* (ATCC 6538) cells in nutrient broth medium (negative control); (**H**) *S. aureus* (ATCC 6538) cells treated with nisin at 5 µg mL^−1^ (positive control) (**I**) *S. aureus* (ATCC 6538) cells treated with ZnO at IC_100_; (**J**) *B. subtilis* (ATCC 19659) cells in nutrient broth medium (negative control); (**K**) *B. subtilis* (ATCC 19659) cells treated with nisin at 5 µg mL^−1^ (positive control); (**L**) *B. subtilis* (ATCC 19659) cells treated with ZnO at IC_100_. Scale bar 2 μm; × 100 magnification.

The integrity of the membranes of *E. coli*, *P. aeruginosa*, *S. aureus*, and *B. subtilis* cells was investigated upon compound exposure using fluorescence microscopy. The results showed the disruption of cytoplasmic membranes in all strains after 15 min of exposure at IC_100_ (Fig. 6). The filters Tx Red and DAPI Blue were applied together and used to visualize PI and DAPI. Cells with intact membranes are artificially stained in blue, while cells with damaged membranes are stained red^35^. Thus, an increase in red-stained cells by PI is related to the increase in cell permeability due to damaged membranes.

In this study, all the bacterial species had their cytoplasmic membrane affected within the first 15 min of exposure to ZnO NPs. Treatment with the compound led to membrane damage in more than 70% of the cells. These results were expected since the bactericidal activity of ZnO NPs was already known, and its predominant MOA is associated with the cell membrane^14^.

ZnO is a transition metal oxide and semiconductor (which belongs to class II–VI) with wide band gap (3.3 eV), there is a general pattern expected. When the radiation has energy larger than the band gap of the ZnO, electron–hole pairs are formed. Electrons are promoted to the conduction band (CB). The hole generated in the valence band (VB) gets a strongly oxidizing character and oxidizing sites are created, which are capable of oxidizing water molecules or hydroxide anions and generate strong oxidizing species^13^. This reaction leads to the redox chain reaction with the generation of reactive oxygen species (ROS) formed by hydroxyl radical (^·^OH), hydroperoxyde radical (^·^HO_2_^−^) and superoxide radical anion (O_2_^·−^) as the pathways of bactericidal action^36^.

Oxidative stress in the bacterial cell can be induced by ROS generation produced from ZnO NPs, which leads to the inhibition of protein synthesis and DNA replication^14^. In this situation, the ZnO conductivity increases, close to the “band gap” of the UV-spectrum characterized by high emission energy. The electronic excitation can destabilize the charges present in the cytoplasmic membrane resulting in their rupture. ZnO can also damage the cytoplasmic membrane by releasing Zn^2+^ ions from the dissolution of ZnO in aqueous solution. The Zn^2+^ ion acts as an inhibitor of the glycolytic enzyme through the thiol group oxidation due to specific affinity for the sulphur group^3^.

**Figure 7:**
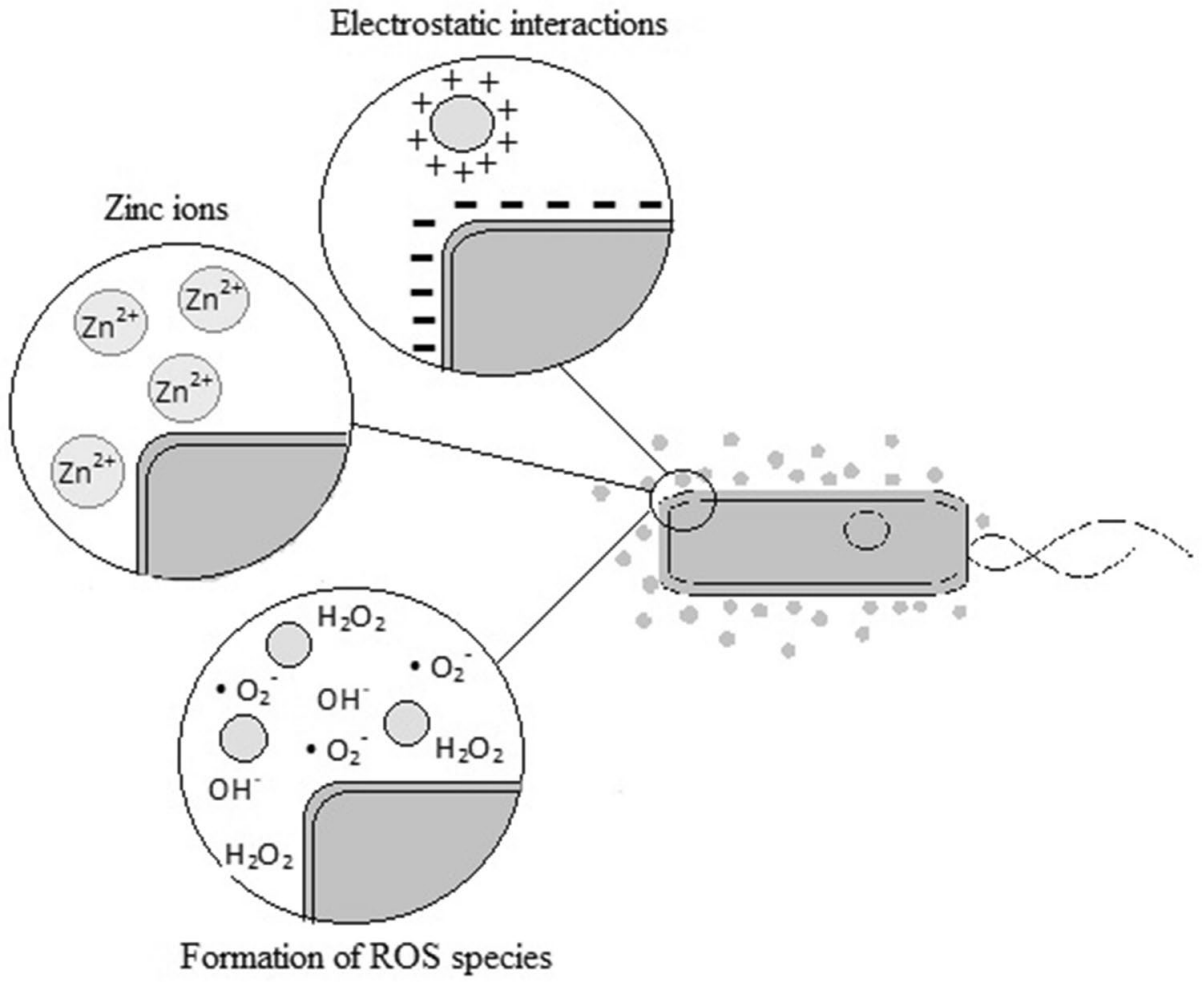
Model of the main bactericidal MOA of ZnO NPs which target the cytoplasmatic membrane and cell wall.

The MOA reported in this study are represented in schematic drawing shown in Fig. 7.

**Figure 8:**
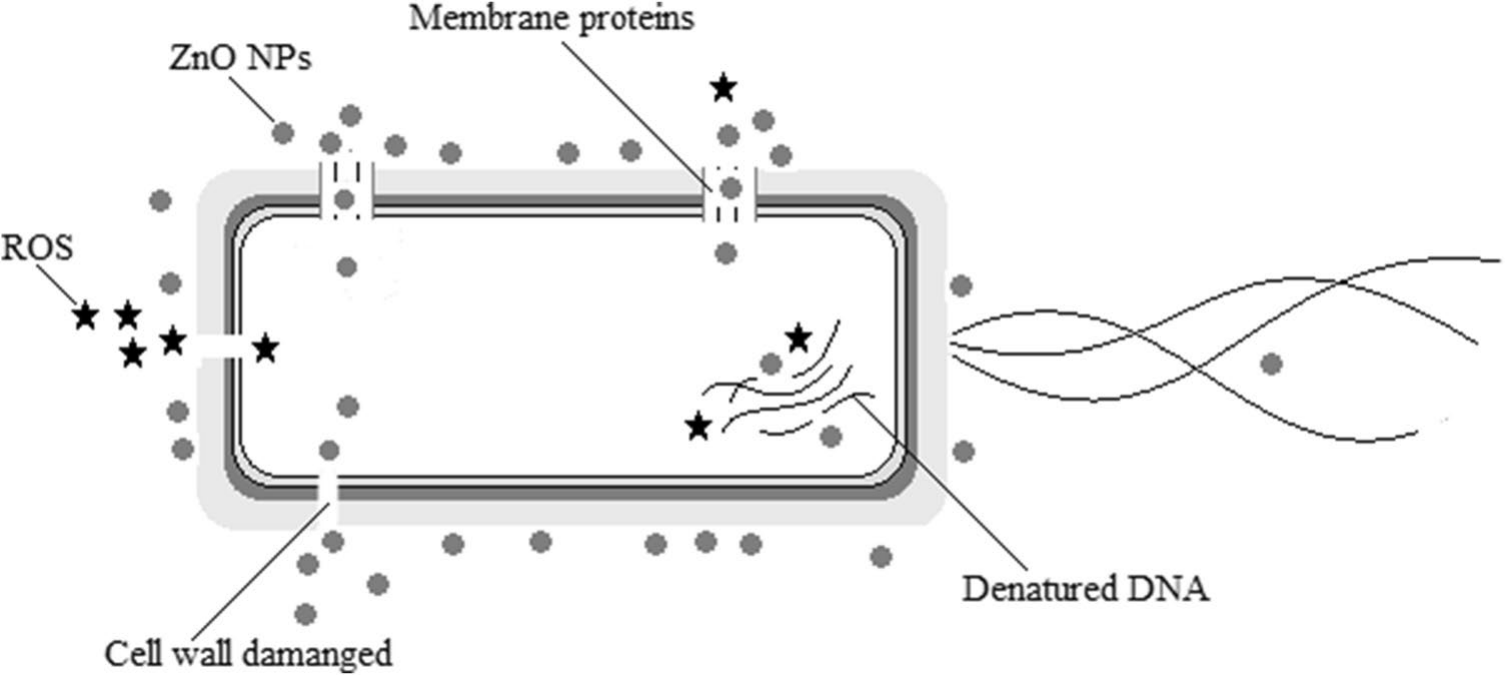
Cell model for the main mechanism of bactericidal action of ZnO NPs.

ZnO NPs can be attached to the surfaces of Gram-positive and Gram-negative bacteria through different pathways. The teichoic acid in the peptidoglycan layer and the lipoteichoic acid in the membrane are the source of negative charges in the cell surface. Positive charges from ZnO NPs are attracted to the cell surface by electrostatic interactions, and the difference in electrostatic gradient leads to damage in the cell surface^37,38^. Teicoic and lipoteichoic acids act as a chelating agent on Zn^2+^ ions, which are then carried by passive diffusion across membrane proteins (Fig. 8). Moreover, the bactericidal action can occur by different mechanisms, such as adsorption in the bacterial surface, formation of different intermediates and electrostatic interactions.

The electrochemical gradient is generated by the movement of hydrogen ions across the cell membrane, which facilitates the diffusion of metallic ions^36^. This mechanism is associated with the size of the material, whose small particles would have better electrostatic interactions. Thus, the ZnO target for inhibitory action is dependent on different factors such as concentration, size and time of interaction.

Zanet et al.^7^ showed that ZnO NPs affect the cell morphology and DNA. However, this can be a side effect, since the main target of ZnO NPs ends up being the first structure they have contact with and consequently act, such as the cytoplasmic membrane. Siddiqi et al.^13^ through SEM and TEM analysis concluded that ZnO NPs damage the cell membrane, and right after go to the cytoplasm, where they interact with other cell structures. Our results also showed damage to the cell. Therefore, it can be concluded that ZnO NPs are multi-target compounds and affect several structures of bacteria cells, but their main mechanism of action is in the cytoplasmic membrane, being other structure effects a consequence/secondary effect after the membrane rupture.
